# Human serum albumin (HSA) regulates the expression of histone-like nucleoid structure protein (H-NS) in *Acinetobacter baumannii*

**DOI:** 10.1038/s41598-022-19012-y

**Published:** 2022-08-27

**Authors:** Jenny Escalante, Brent Nishimura, Marisel R. Tuttobene, Tomás Subils, Camila Pimentel, Nardin Georgeos, Rodrigo Sieira, Robert A. Bonomo, Marcelo E. Tolmasky, Maria Soledad Ramirez

**Affiliations:** 1 Center for Applied Biotechnology Studies, Department of Biological Science, College of Natural Sciences and Mathematics, California State University Fullerton, 800 N State College Blvd, Fullerton, CA 92831 USA; 2 Área Biología Molecular, Facultad de Ciencias Bioquímicas y Farmacéuticas, Universidad Nacional de Rosario, Rosario, Argentina; 3 Instituto de Biología Molecular y Celular de Rosario (IBR, CONICET-UNR), Rosario, Argentina; 4 Instituto de Procesos Biotecnológicos y Químicos de Rosario (IPROBYQ, CONICET-UNR), Rosario, Argentina; 5 Fundación Instituto Leloir – IIBBA CONICET, Buenos Aires, Argentina; 6 Research Service and GRECC, Louis Stokes Cleveland Department of VeteransAffairs Medical Center, Cleveland, OH USA; 7 Departments of Medicine, Pharmacology, Molecular Biology and Microbiology, Biochemistry, Proteomics and Bioinformatics, Case Western Reserve University School of Medicine, Cleveland, OH USA; 8 CWRU-Cleveland VAMC Center for Antimicrobial Resistance and Epidemiology (Case VA CARES), Cleveland, OH USA

**Keywords:** Microbiology, Pathogenesis

## Abstract

According to the Centers for Disease Control and Prevention, *Acinetobacter baumannii* is listed among the most threatening pathogens. *A. baumannii* is mainly a nosocomial pathogen with a distinctive ability to survive in multiple environments. These characteristics together with this bacterium’s ability to acquire antibiotic resistance determinants make it a notorious pathogen. The presence of human serum albumin (HSA) is associated with modification of expression levels in numerous genes. The presence of HSA in the culture medium is also correlated with a reduction in levels of the global suppressor histone-like nucleoid structure protein, H-NS. Comparative transcriptome analysis of the wild type and isogenic *Δhns* strains cultured in lysogeny broth (LB) in the presence or absence of HSA revealed that the expression of a subset of eleven genes are modified in the *Δhns* cultured in LB and the wild-type strain in the presence of HSA, pointing out these genes as candidates to be regulated by the presence of HSA through H-NS. Six and five of these genes were up- or down-regulated, respectively. Three of these genes have functions in quorum sensing (*acdA, kar* and *fadD*), one in quorum quenching (*aidA*), two in stress response (*katE, ywrO*)*,* three in metabolism (*phaC*, *yedL1*, and *yedL2*), one in biofilm formation (*csuAB*), and one in β-oxidation of fatty acids (*fadA*). The regulation of these genes was assessed by: (i) transcriptional analysis and qPCR at the transcriptional level; and (ii) by determining the phenotypic characteristics of each function. The results of these studies support the hypothesis that HSA-mediated reduction of H-NS levels may be one very important regulatory circuit utilized by *A. baumannii* to adapt to selected environments, such as those where HSA-containing human fluids are abundant.

## Introduction

*Acinetobacter baumannii* has emerged as a significant nosocomial pathogen as it is associated with high levels of morbidity and mortality^1–5^. The majority of strains recovered from patents in the hospital are multidrug resistant (MDR), a characteristic that permitted the World Health Organization (WHO) and the Centers for Disease Control and Prevention (CDC) to classify this pathogen as an “urgent threat”^6,7^.

*A. baumannii* strains are genetically diverse as a result of their extraordinary capacity to naturally acquire DNA via transformation^8–13^. Recent studies show that human serum albumin (HSA), a major blood protein, enhances transformation frequency and increases expression levels of type IV pilus-associated genes in *A. baumannii*^9,14,15^. Moreover, the effect of HSA is not limited to these functions; HSA also affects expression of genes involved in motility, biofilm formation, efflux pumps, metabolism, capsule synthesis, transcriptional regulation, antibiotic resistance, and pathogenesis as determined by transcriptomic analysis^14^. These studies also reveal that the *A. baumannii h-ns* (*h*istone-like *n*ucleoid *s*tructuring) gene expression levels decrease in cells exposed to purified HSA. An attractive hypothesis to explain a physiological role from these observations is that there is a regulatory circuit that links HSA to reduced H-NS protein intracellular concentrations, which affect expression of selected genes helping *A. baumannii’*s adaptation to HSA-containing human fluids^14^.

In *A. baumannii* the H-NS protein, a global transcriptional repressor in many Gram-negative bacteria^16–19^, plays a major role in persistence and expression of virulence-associated genes^17,20^, stress induced by carbapenemase expression^21^, as well as expression of genes associated with antibiotic resistance, biofilm formation, and quorum sensing^22^. H-NS may also be necessary for natural competence in *A. baumannii* as an *hns* null mutant showed significantly lower expression levels of genes related to acquisition of DNA from the environment^23^. To better understand the correlation of the effects produced by extracellular HSA and intracellular H-NS, we studied the effect of the presence of HSA in the milieu on the expression H-NS and other *A. baumannii*’s genes. Comparative transcriptomic analyses (RNA-seq) using *A. baumannii* AB5075 and AB5075 *Δhns* cultured in the presence or absence of HSA revealed significant modifications in levels of expression of specific genes. These experiments, in combination with phenotypic assays, indicate that HSA modifies expression levels of some *A. baumannii* genes through regulating expression of H-NS.

## Results and discussion

### Transcriptional effect of HSA on gene expression

**Figure 1:**
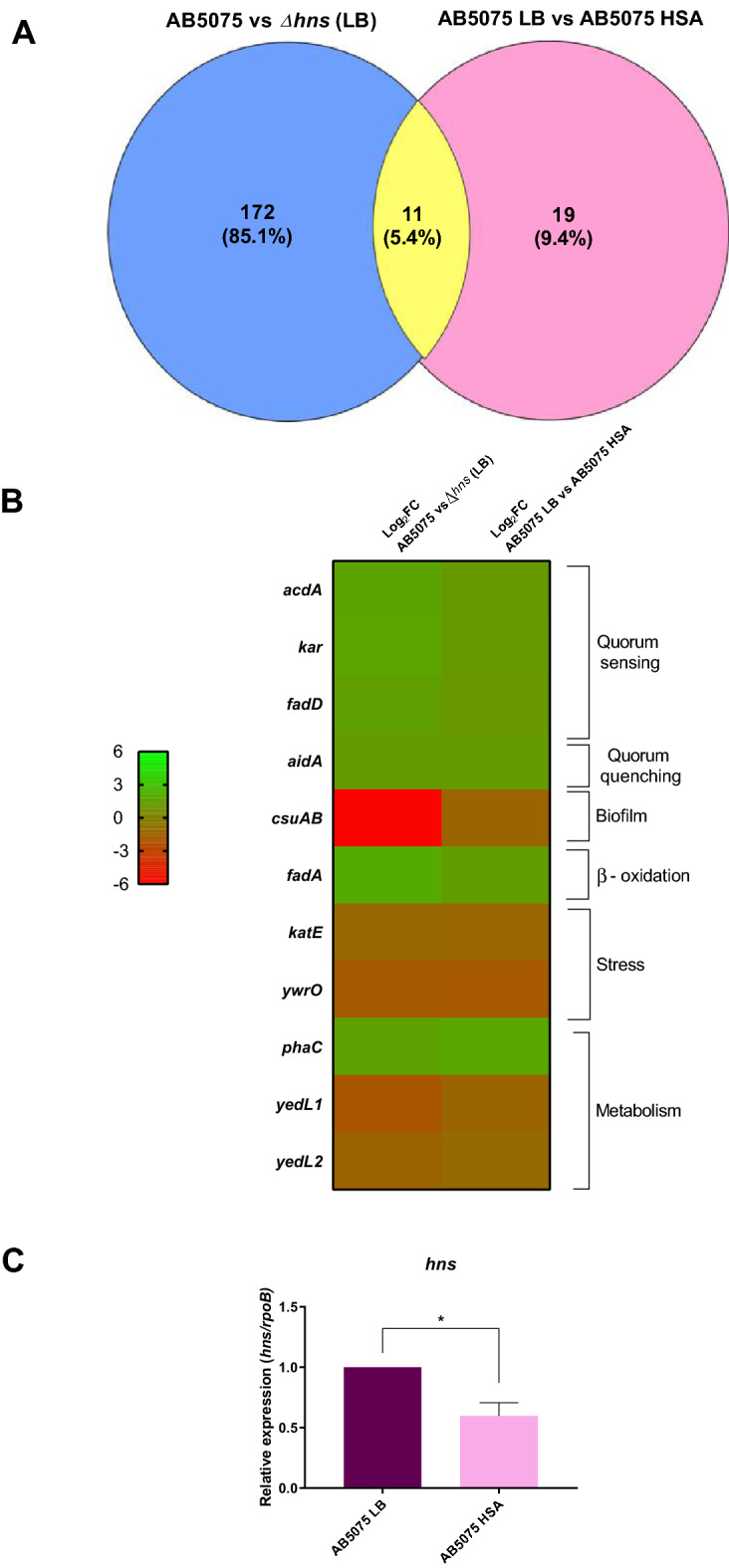
(**A**) Venn diagram shown the transcriptional analysis comparison between *A. baumannii AB5075* vs *A. baumannii Δhns* (both strains cultured in LB broth) and AB5075 (cultured in LB broth) vs *A. baumannii* AB5075 (cultured in LB broth supplemented with 3.5% HSA). (**B**) Heat-map shown the total of 11 DEGs (hhDGEs) found in common between the two analyses. The scale goes from − 6 to 6. (**C**) qRT-PCR analysis of *hns* of *A. baumannii* AB5075 cultured in LB broth vs AB5075 cultured in LB broth supplemented with 3.5% HSA. Fold changes were calculated using double ΔCt analysis. At least three independent samples were used, and four technical replicates were performed from each sample. Student’s *t* test analysis was performed using GraphPad Prism (GraphPad software, San Diego, CA, USA). A *P* < 0.05 was considered significant. Data are presented as mean ± SD.

Transcriptome analysis of *A. baumannii* AB5075 and AB5075 *Δhns* cultured in LB broth revealed a differential expression profile of 183 genes^22^. When *A. baumannii* AB5075 was cultured in LB broth with or without HSA there were 30 genes differentially expressed (Table S1). In both cases, an FDR-adjusted *P value* of < 0.05 and log_2_-fold change > 1 was considered as the differentially expressed-genes (DEGs). Inspection of the DEGs in both analyses showed that there were 11 genes in common (hhDEG) (Fig. 1A and Table S2). Among the eleven genes, six (*acdA, kar*, *fadD, aidA, fadA,* and *phaC*), and five (*csuAB*, *katE, ywrO, yedL1*, and *yedL2*) of these DEGs were up- or down-regulated, respectively (Fig. 1B). Three of these genes are involved in quorum sensing (*acdA, kar* and *fadD*), and one in quorum quenching (*aidA*)^24^. Other hhDEGs are associated with biofilm formation (*csuAB*)^25^, β-oxidation of fatty acid (*fadA*)^26^, stress response (*katE, ywrO*)^27^, and metabolism (*phaC*, *yedL1*, and *yedL2*) (Fig. 1B). Quinn et al. previous observation^14^, where H-NS is modified in the presence of HSA, together with genes known to be regulated by H-NS, and our present transcriptomic comparison (AB5075 vs Δ*hns* (both strains cultured in LB broth), vs, AB5075 (cultured in LB broth) vs AB5075 (cultured in 3.5% HSA), point out these eleven genes as candidates to be regulated by the presence of HSA through modification of the intracellular H-NS concentration.

Cultures of the multidrug resistant, hypervirulent *A. baumannii* AB5075 model strain in the presence or absence of a physiological concentration of HSA were next subjected to quantitative RT-PCR (qRT-PCR) using total RNA to confirm that the presence of HSA in the growth medium results in a reduction of the H-NS synthesis (Fig. 1C)*.*

**Figure 2:**
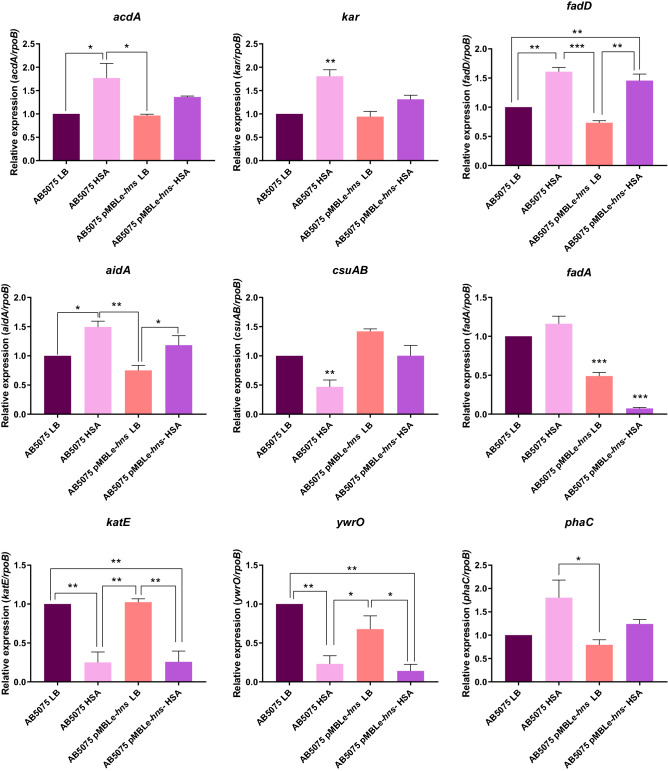
Genetic analysis of genes of *A. baumannii* AB5075 or AB5075 pMBLe-*hns* cultured in LB broth or LB broth supplemented with 3.5% HSA. qRT-PCR of genes associated with quorum sensing, *acdA, kar*, and *fadD,* with quorum quenching*, aidA,* with biofilm*, csuAB,* with β-oxidation*, fadA,* with stress*, katE and ywrO* and metabolism *phaC* expressed in LB or LB supplemented with HSA. Fold changes were calculated using double ΔCt analysis. At least three independent samples were used, and four technical replicates were performed from each sample. The *A. baumannii* AB5075 cultured in LB was used as reference. Data are presented as mean ± SD. Statistical significance (*P* < 0.05) was determined by ANOVA followed by Tukey’s multiple-comparison test, one asterisks: *P* < 0.05; two asterisks: *P* < 0.01 and three asterisks: *P* < 0.001. This figure was performed using GraphPad Prism version number 9 (GraphPad software, San Diego, CA, USA, https://www.graphpad.com/).

We next determined the levels of H-NS in *A. baumannii* AB5075 pMBLe-*hns*, a strain that carries a plasmid that overexpresses H-NS. Figure S1 shows that expression was elevated in cells grown in LB medium, but the H-NS levels were reduced fourfold when the medium was supplemented with 3.5% HSA. This strain and the wild type *A. baumannii* AB5075 were used to assess the expression of nine selected hhDEGs by qRT-PCR using RNA obtained from cultures carried out in LB broth supplemented with and without 3.5% HSA. Expression of *acdA, kar, fadD,* and *aidA* were upregulated about twofold in *A. baumannii* AB5075 grown in the presence of HSA. However, it was of interest that increasing the concentration of H-NS did not modify the expression of these genes (compare bar AB5075LB with AB5075 pMBLe-*hns* in Fig. 2). Expression of *csuAB* was downregulated twofold in *A. baumannii* AB5075 growing in the presence of HSA.

In contrast, the *fadA* gene behaved unexpectedly; significant changes were not observed in the presence or absence of HSA in *A. baumannii* AB5075 and a tenfold reduction in expression was observed in the overproducing strain that was driven by the presence of HSA, i.e., when overexpression of H-NS was reversed (Fig. 2). The molecular basis of these modifications and changes are unknown at this time. The stress-response related genes *katE* and *ywrO* were downregulated around threefold in the presence of HSA and differences were not noted when H-NS was overexpressed (Fig. 2). The metabolic gene *phaC* a key enzyme in the polymerization of polyhydroxyalkanoates (PHAs), showed a significant twofold up-regulation in both strains, but significant modifications to levels of expression when H-NS was overexpressed was not seen (Fig. 2).

Since biofilm and quorum sensing are intimately related to *A. baumannii’s* virulence (and we identified genes involved in these two processes that are regulated by HSA and H-NS), we expanded the qRT-PCR analysis to two other genes associated with these processes. The expression of *csuE,* associated with biofilm formation, was downregulated about twofold when *A. baumannii* AB5075 was cultured in the presence of HSA. In *A. baumannii* AB5075 pMBLe-*hns* was overexpressed; but a significant downregulation was observed when HSA was present in the culture medium (Fig. S2). In the case of *abaI*, which codes for the acyl homoserine lactone (AHL) synthase, the presence of HSA in the milieu produced a significant threefold reduction in expression. *A. baumannii* AB5075 pMBLe of H-NS growing in the presence or absence of HSA expressed the gene at low levels (Fig. S2).

### HSA plays a role in modulating the biofilm formation, the quorum sensing network, and the oxidative stress through H-NS

**Figure 3:**
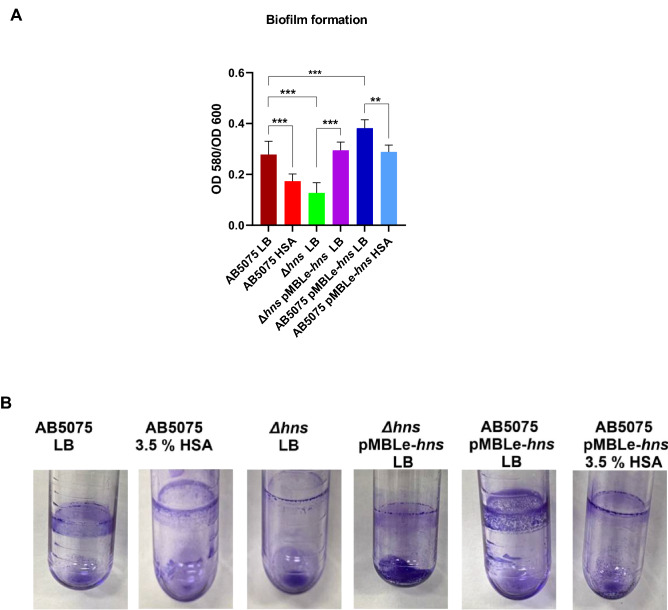
Phenotypic analysis of biofilm formation. Biofilm assays performed with the AB5075 strain grown in LB or LB supplemented with 3.5% HSA, *Δhns* and *Δhns* pMBLe-*hns* strains cultured in LB, and AB5075 pMBLe-*hns* culture in LB or LB supplemented with 3.5% HSA. (**A**) Quantification of the radio of biofilm to total biomass in polystyrene wells. The mean ± SD is informed of three independent experiments. Statistical significance (*P* < 0.05) was determined by ANOVA followed by Tukey’s multiple comparison test, one asterisks: *P* < 0.05; two asterisks: *P* < 0.01 and three asterisks: *P* < 0.001. (**B**) Tubes of biofilm experiment after the stained with 1% crystal violet y removed the excess. A representative experiment is shown. This figure was performed using GraphPad Prism version number 9 (GraphPad software, San Diego, CA, USA, https://www.graphpad.com/).

*A. baumannii* AB5075 grown in the presence of HSA and the *hns* deficient mutant strain produced a reduced mass of biofilm in comparison to the wild type when the biofilm production was quantified using a previously described method in polystyrene wells (Fig. 3A). Similar results were observed when the biofilm formation was assessed in tubes (Fig. 3B). The *A. baumannii* AB5075 pMBLe-*hns* strain as well as the mutant complemented by the plasmid pMBLe-*hns* cultured in the absence of HSA showed elevated levels of biofilm production. In *A. baumannii* AB5075 pMBLe-*hns* a regulatory effect was noted in HSA-containing medium. Taken together, these results support a regulatory role of HSA through modifying H-NS expression.

Phenotypic modifications in quorum sensing were assessed determining levels of acyl-homoserine lactone (AHL) using the *Agrobacterium tumefaciens*-based solid plate assays^28,29^. Rodgers et al. demonstrated that the supernatants from *A. baumannii* AB5075 or AB5075 *Δhns* produced similar intensity of color, likely caused by an increase in both AHL synthesis (quorum sensing) and lactonase activity (quorum quenching) in AB5075 *Δhns* strain^22^. To study the effect of HSA on AHL secretion into the surrounding medium, *A. baumannii* AB5075, AB5075 *Δhns,* and the strains complemented with pMBLe-*hns* were cultured in different conditions before assessing the AHL concentration in the growth medium. *A. baumannii* AB5075 produced much lower amount of AHL when it was cultured in the presence of HSA (Fig. S3). *A. baumannii* AB5075 pMBLe-*hns* growing in the presence or absence of HSA and *A. baumannii* AB5075 *Δhns* pMBLe-*hns* cultured in LB broth, all produced low levels of AHL. The low AHL concentration in the growth medium could be explained by cancelling modifications in expression of genes associated with AHL synthesis (*acdA, kar, fadD,* and *abaI*) and lactonase, which is responsible for quorum quenching activity (*aidA*) (Figs. 1, 2, and S2).

**Figure 4:**
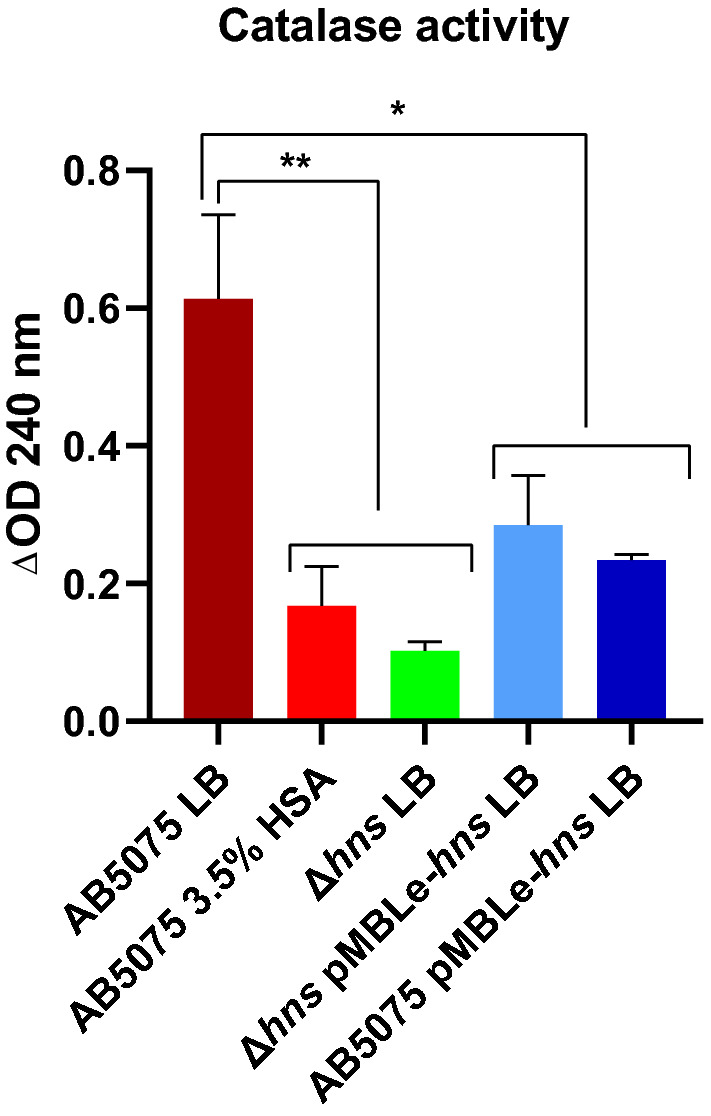
Phenotypic analysis of catalase activity. Monitoring the decrease in absorbance at 240 nm of crude extract of *A. baumannii* AB5075 and derivate strains cultured in LB or LB supplemented with 3.5% HSA. The mean ± SD is informed of three independent experiments. Statistical significance (*P* < 0.05) was determined by ANOVA followed by Tukey’s multiple comparison test, one asterisks: *P* < 0.05; two asterisks: *P* < 0.01 and three asterisks: *P* < 0.001. This figure was performed using GraphPad Prism version number 9 (GraphPad software, San Diego, CA, USA, https://www.graphpad.com/).

Hydrogen peroxide is a disinfectant with potent bactericidal activity that is used in vaporized form to control outbreaks of multi-resistant *A. baumannii* infections^30–32^. Since *A. baumannii* is catalase-positive and the *katE* gene was a hhDEG, down-regulated in the presence of HSA, we assessed the production of catalase activity, as the decrease in absorbance at 240 nm resulting from the consumption of H_2_O_2_ (Fig. 4). Addition of HSA to the growth medium resulted in a reduction of catalase activity in the wild type strain. Equally low activity was produced by *A. baumannii* AB5075 *Δhns* growing in the absence of added HSA, which suggest that the action of HSA occurs through reduction of H-NS synthesis (Fig. 4). These results agreed with both RNA-seq analyses and RT-qPCR results. As expected, the complemented *A. baumannii* AB5075 *Δhns* pMBLe-*hns* produced higher catalase activity when compared to *A. baumannii* AB5075 (Fig. 4). Surprisingly, overexpressing *hns* showed reduced catalase activity when compared to the wild type strain; we still do not know the molecular bases for this unexpected results (Fig. 4).

The results described in this section support the hypothesis that some *A. baumannii* genes are regulated by H-NS, whose expression is modified by the presence of HSA in the milieu. H-NS is a nucleoid-associated protein that binds DNA in a relatively nonspecific manner (it shows preference for AT-rich and curved regions) and alters its topology, which modifies levels of transcription. The effects of H-NS in *A. baumannii* have not been thoroughly studied^17,20,22^. An early analysis found that an *A. baumannii* mutant containing a disrupted *hns* gene was modified in the expression of several virulence genes such as those associated with the autotransporter Ata, the type VI secretion system, a type I pilus cluster, the acetoin metabolism, and phenylacetic acid degradation^17^. This strain also showed altered adherence to biotic surfaces and increase virulence as determined using the *Caenorhabditis elegans* infection model system^17^. A previous study showed that exposure of the model *A. baumannii* A118 to HSA was accompanied by a reduction of expression of H-NS and a wide variety of genes related to antibiotic resistance and stress response as well as genes that encode functions associated with natural competence^14,22,23,33^.

The global nature of H-NS as transcriptional regulator makes it challenging to definitely associate genes that are regulated by H-NS through variations in levels of expression of the regulator when the cells are exposed to HSA. However, despite these limitations, the results described in this work strongly suggest that there is a group of genes whose expression is indirectly regulated at the transcriptional level by HSA through reducing H-NS expression. We conclude that HSA-mediated reduction of H-NS levels may be one regulatory circuit utilized by *A. baumannii* to adapt to selected environments such as those where HSA-containing human fluids are abundant.

## Conclusions

H-NS is a highly abundant intracellular protein that functions as a nucleoid organizer and a transcriptional silencer. In this work we gathered evidence that supports the role of HSA and H-NS in regulating quorum sensing and quorum quenching, biofilm, β-oxidation, stress, and metabolism related genes. Future analysis planned using chromatin immunoprecipitation followed by high-throughput sequencing (ChIP-seq), in wild-type or an *h-ns* mutant, in the presence or absence of HSA will contribute to new insights into the molecular mechanisms governing the roles of HSA and H-NS in regulating the genes identified in this work and most probably other genes involved in the mechanisms of persistence and pathogenicity of *A. baumannii*.

## Materials and methods

### Bacterial strains

The multidrug and hypervirulent AB5075 strain^34^, its isogenic *hns* mutant (AB5075*Δhns*)^35^ and AB5075 *Δhns*pMBLe-*hns*^22^ were used in the present study.

### Electroporation

Electro-competent *A. baumannii* AB5075 cells were prepared and mixed with pMBLe-*hns* plasmid DNA (containing apramycin resistance) followed by electroporation with a Bio-RadGene Pulser instrument as described previously^21^. The electroporated cells were placed in recovery in a shaking incubator followed by culturing overnight at 37 °C on LB agar containing15 μg/ml apramycin (*aac(3)-IV*). At least 10 colonies were picked to confirm the presence of the different plasmids. To confirm their presence, plasmid extraction followed by gel electrophoresis analysis.

### RNA extraction and RNA-seq analysis

*A. baumannii* AB5075 and derivative strains were cultured in LB broth and incubated with agitation for 18 h at 37 °C. Overnight cultures were then diluted 1:10 in fresh LB broth or LB broth supplemented with HSA and incubated with agitation for 7 h at 37 °C. The Direct-zol RNA Kit (Zymo Research, Irvine, CA, USA) was used to perform the RNA extraction in triplicates. RNA samples were DNase treated (Thermo Fisher Scientific, Waltham, MA, USA) following manufacturer’s instruction. Samples were confirmed to have no DNA contamination through PCR amplification of the 16S rDNA gene. RNA sequencing was outsourced to Novogene (Novogene Corporation, Sacramento, CA, USA). Ribosomal RNA-depletion was done using the Ribo-Zero kit (Illumina) and the construction of the cDNA library was performed with the TruSeq Stranded Total RNA Library Prep kit (Illumina) from three independent replicates per sample. Analysis of the quality of the Illumina reads, trimming of low-quality bases and removal of Illumina adapters was performed as described previously^36^. Reads were aligned to the genome of *A. baumannii* AB5075 using Burrows-Wheeler Alignment (BWA) software (v0.7.17) BWA and visualized using the Integrative Genomics Viewer (IGV). Read counts per gene were calculated using FeatureCounts^37^, and differential expression analysis was performed using DEseq2.Differentially expressed genes (DEGs) were defined as those displaying an FDR adjusted *P value* of < 0.05 and log2 fold change > 1. In the present work, previously published RNA-seq reads (GEO accession No GSE167117) corresponding to *A. baumannii* AB5075 vs AB5075 *Δhns* incubated in LB broth^22^ or *A. baumannii* AB5075 grown in LB broth vs grown in 3.5% HSA were analyzed.

qRT-PCR was performed to confirm and analyze the expression genes. cDNAwas prepared using the iScript Reverse Transcription Supermix for qRT-PCR (BioRad, Hercules, CA, USA) and quantitative PCR was performed using iQ SYBR Green Supermix (BioRad, Hercules, CA, USA) per the manufacturer’s recommendations, respectively. Results were analyzed using the 2^−ΔΔCt^ method^38^ in which *rpoB* was used as the control gene. *rpoB* genes was confirmed to be a suitable reference gene showing a stable expression under the tested conditions using four other additional genes (16S rRNA, *recA*, *secA* and *gyrB*) per comparison. Experiments were performed in technical and biological triplicates. The results from experiments performed were statistical analyzed (ANOVA followed by Tukey’s multiple comparison test) using GraphPad Prism (GraphPad software, San Diego, CA, USA). A *P value* < 0.05 was considered significant.

The RNA-seq data analysed during the current study are available in the NCBI repository with the GEO accession No GSE167117.

### Catalase activity measurements

Crude extract of *A. baumannii* AB5075 and derivate strains cultured in LB or LB supplemented with 3.5% HSA were utilized to determinate the catalase activity spectrophotometrically by monitoring the decrease in absorbance at 240 nm resulting from the consumption of H_2_O_2_ using a UV visible spectrophotometer (SpectraMax M3)^39^.

### Biofilm assay

*A. baumannii* AB5075 and derivative strains were cultured in fresh LB medium, or LB supplemented with 3.5% HSA with agitation for 18 h at 37 °C. Tubes were emptied, washed three times with 1X phosphate-buffered saline (PBS) and stained with 1% crystal violet (CV) for 15 m. Excess CV was removed by washing three more with 1X PBS. In addition, quantification of biofilm production in polystyrene wells were carried out using a protocol from previously described method^14^. Experiments were performed in triplicate, with at least three technical replicates per biological replicate.

### N-acyl homoserine lactone (AHL) detection

*Agrobacterium tumefaciens*-based solid plate assays were carried out to detect N-Acyl Homoserine Lactone (AHL) production^40^ as described in previous work^28^.Initially, 500 μL of the homogenate were loaded in a central well of 0.7% LB agar plates supplemented with 40 μg of 5-bromo-3-indolyl-β-D-galactopyranoside (X-Gal) per mL and 250 μL (OD = 2.5) of the overnight culture of *Agrobacterium tumefaciens* biosensor. The presence of AHL was determined by the development of blue coloring^29^. As a positive control, 100 μL of N-decanoyl-dl-homoserine lactone (C10-AHL) 12.5 mg/mL was utilized. Quantification of 5,5′-dibromo-4,4′-dichloro-indigo production in different conditions was determined using ImageJ software (NIH) by measuring the intensity of each complete plate, and subtracting the intensity measured in the negative control. The values were normalized to the positive control, which received the arbitrary value of 100.

### Statistical analysis

Experiments performed at least in triplicates were statistically analyzed by ANOVA followed by Tukey’s multiple comparison tests using GraphPad Prism (GraphPad software, San Diego, CA, USA). A *P value* < 0.05 was considered significant.

All procedures performed in this study were in accordance with the CSUF Institutional Biosafety Committee Approval plan (DBH117-01) and follow the NIH, CDC, OSHA and other environmental and occupational regulations.

## Supplementary Information

Supplementary Information.

## Data Availability

The datasets generated and analyzed during the current study are available in the Gene Expression Omnibus (GEO) repository, (GEO accession No GSE167117).
