# Supplementary material for: Human serum albumin (HSA) regulates the expression of histone-like nucleoid structure protein (H-NS) in Acinetobacter baumannii
Source: Sci Rep. 2022 Aug 27;12:14644. doi: 10.1038/s41598-022-19012-y (PMC9420150; doi:10.1038/s41598-022-19012-y)
Supplement: Supplementary file 1 — Supplementary Information. [file 41598_2022_19012_MOESM1_ESM.docx]

**
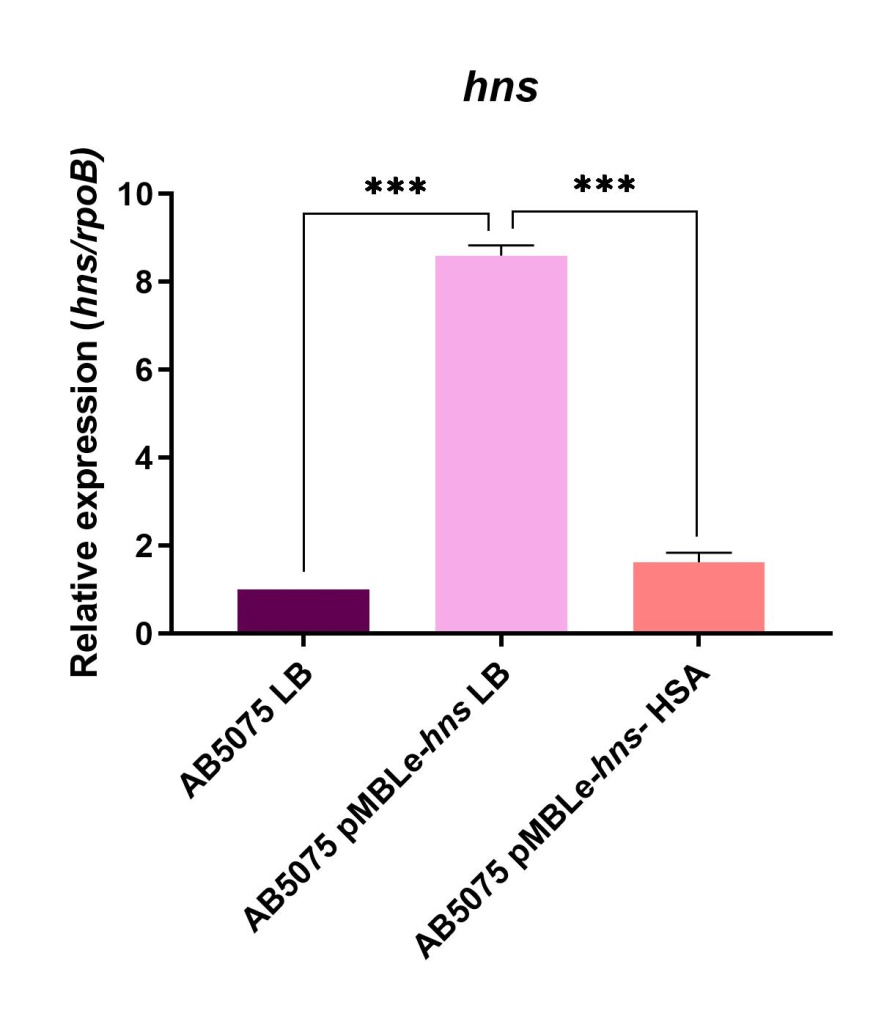
**

**Figure S1.** qRT-PCR analysis of *hns* of *A. baumannii* AB5075 pMBLe-*hns* cultured in LB broth or LB broth supplemented with 3.5 % HSA respect to parental strain. Fold changes were calculated using double ΔCt analysis. At least three independent samples were used, and four technical replicates were performed from each sample. Statistical significance (*P* < 0.05) was determined by ANOVA followed by Tukey’s multiple-comparison test, one asterisks: *P* < 0.05; two asterisks: *P* < 0.01 and three asterisks: *P* < 0.001.


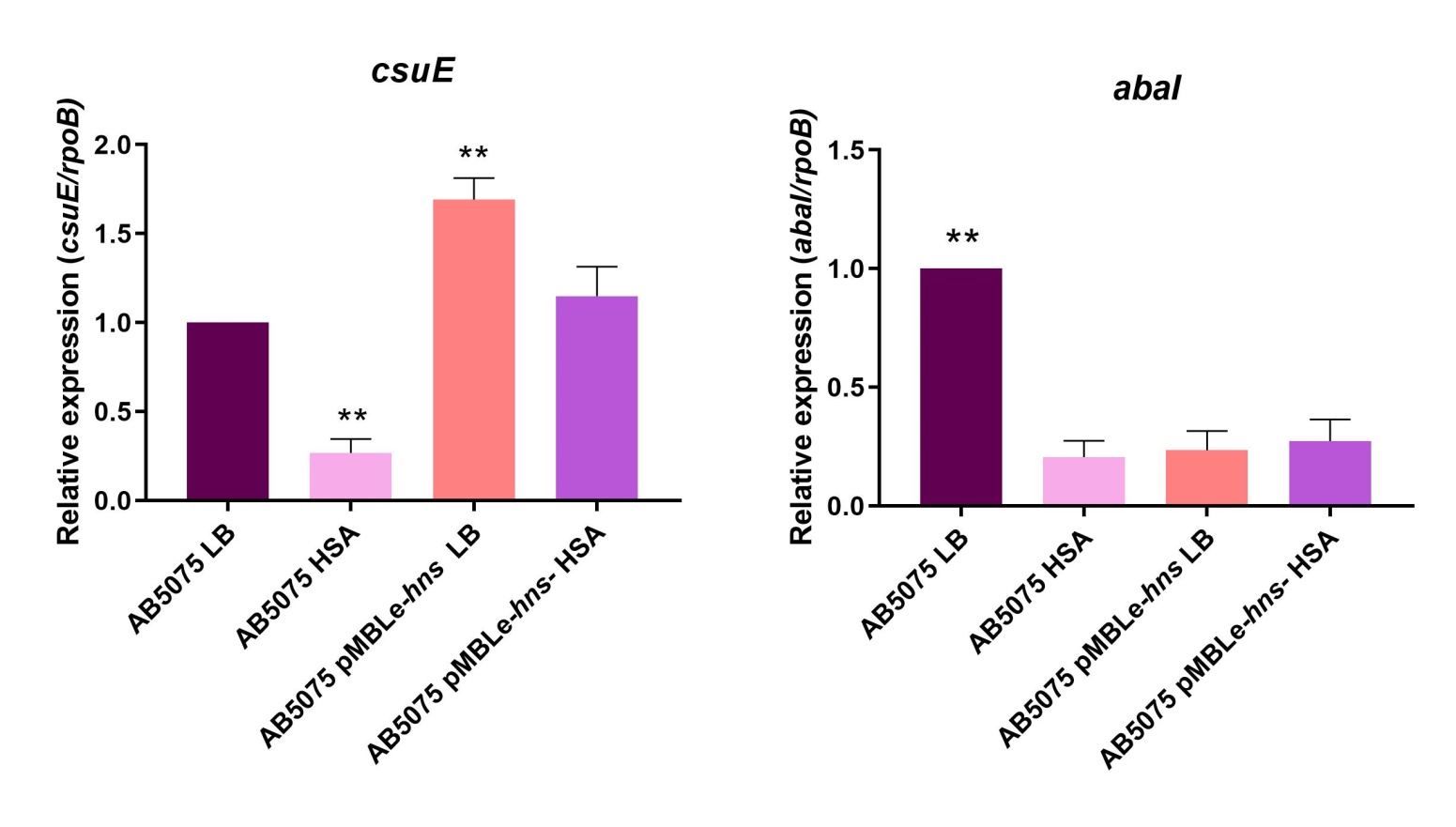


**Figure S2.** qRT-PCR analysis of *csuE and abaI* of *A. baumannii* AB5075 pMBLe-*hns* cultured in LB broth or LB broth supplemented with 3.5 % HSA respect to parental strain. Fold changes were calculated using double ΔCt analysis. At least three independent samples were used, and four technical replicates were performed from each sample. Statistical significance (*P* < 0.05) was determined by ANOVA followed by Tukey’s multiple-comparison test, one asterisks: *P* < 0.05; two asterisks: *P* < 0.01 and three asterisks: *P* < 0.001.


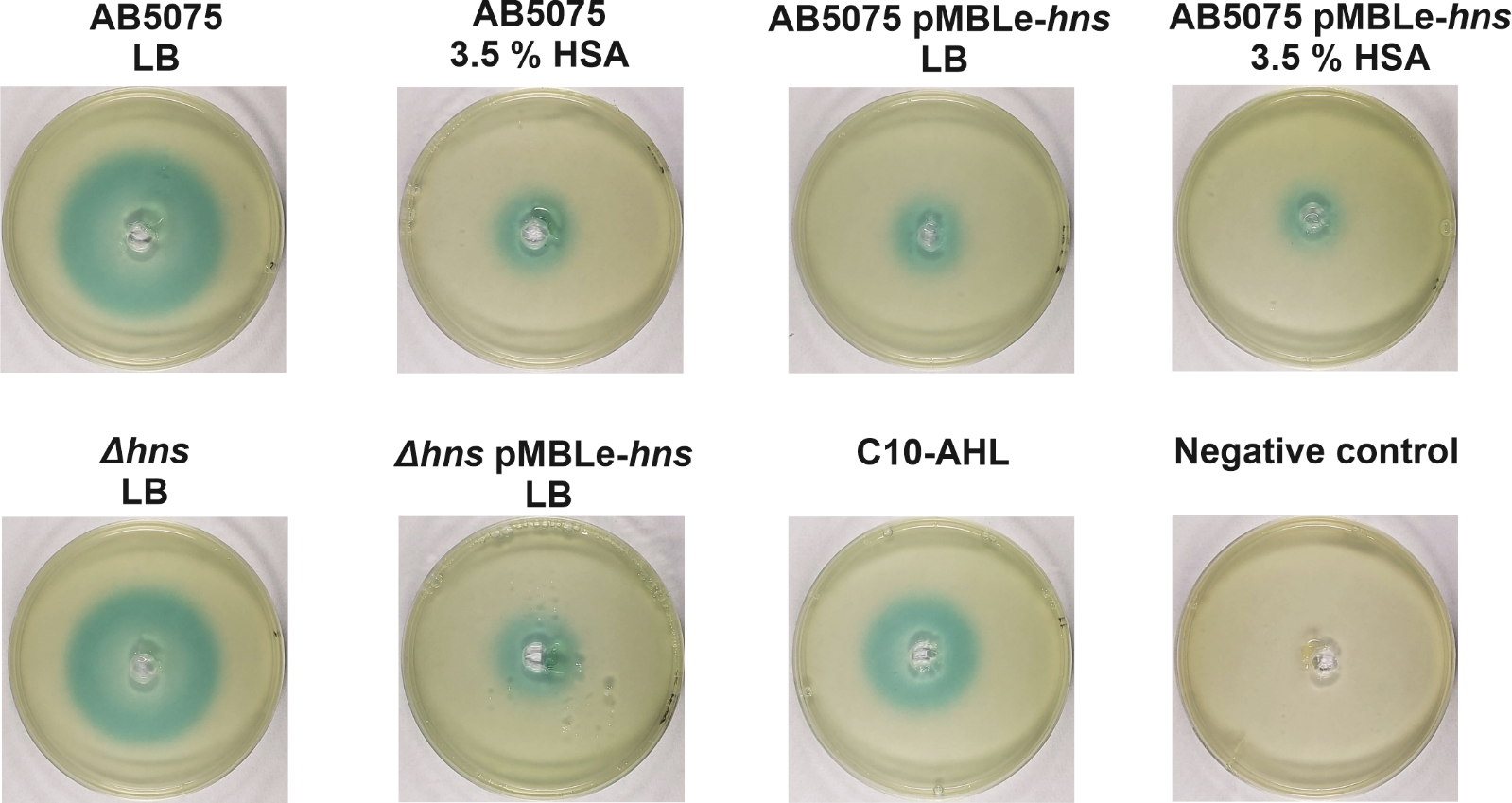


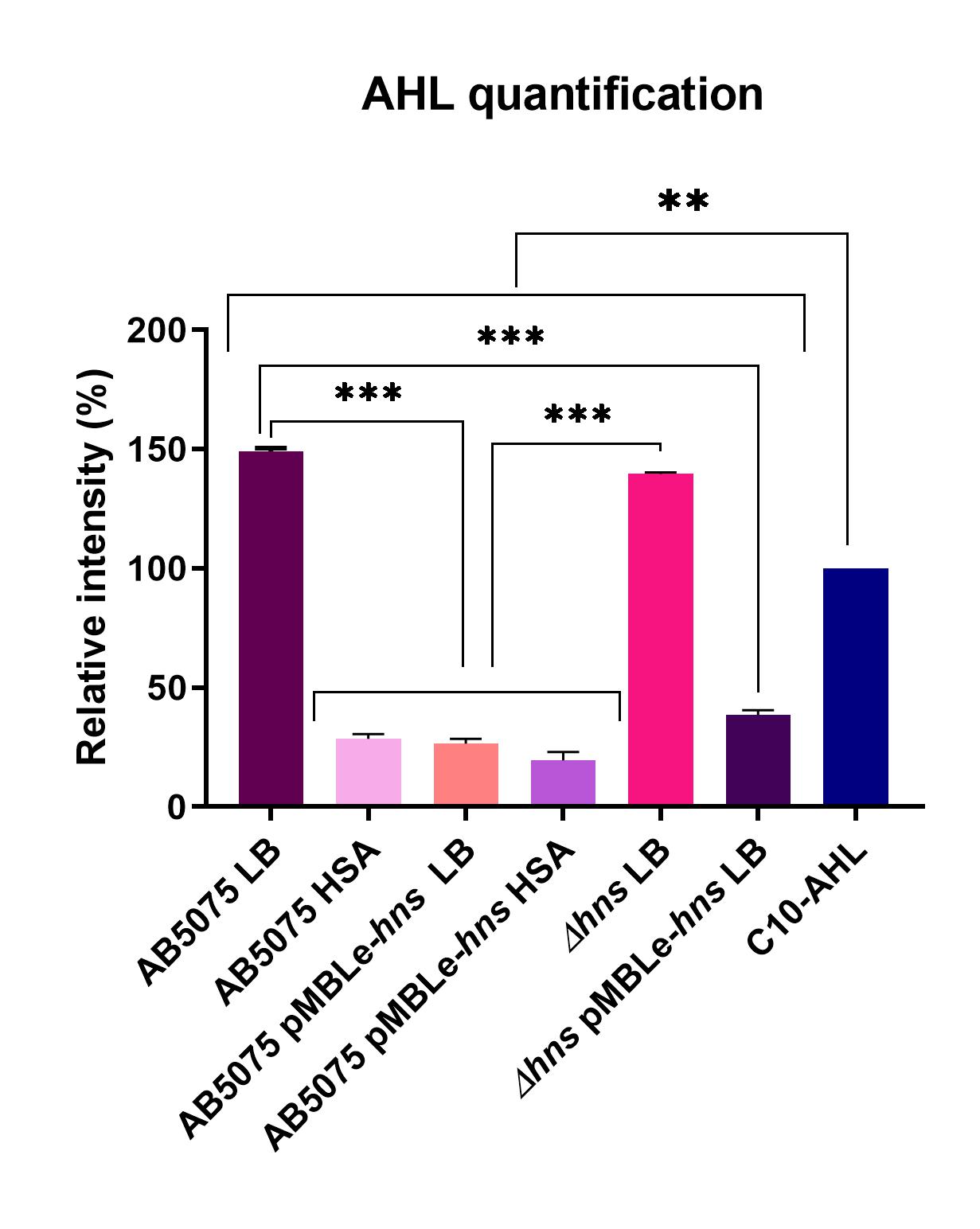


**Figure S3.** Phenotype analysis of quorum network. The presence of AHL was determined by the development of the blue color. Quantification of 5,5′-dibromo-4,4′-dichloro-indigo were estimated as the percentage relative to C10-AHL standard, measured with ImageJ (NIH). The mean ± SD is informed of three independent experiments. Statistical significance (*P* < 0.05) was determined by ANOVA followed by Tukey’s multiplecomparison test. Experiments were performed in triplicate, with at least three technical replicates per biological replicate. This figure was performed using GraphPad Prism version number 9 (GraphPad software, San Diego, CA, USA, https:// www. graph pad. com/).

**Table S1: Transcriptional analysis between *A. baumannii* AB5075 (LB broth) vs *A. baumannii* AB5075 (LB broth supplemented with HSA)**

| LB WT – HSA WT |  | Log2FC | padj |  |
| --- | --- | --- | --- | --- |
| A591_A0345 | *acdA* | 1,1284805 | 0,0417648 | "acyl-CoA dehydrogenase N-terminal domain protein" |
| A591_A0587 | Hly-III family | 1,4060963 | 6,179E-19 | "channel protein, hemolysin III family" |
| A591_A0906 |  | -1,017696 | 2,373E-14 | "outer membrane efflux protein" |
| A591_A1044 |  | 1,7806483 | 9,463E-16 | "oxidoreductase NAD-binding domain protein" |
| A591_A1045 | *scd* | 1,7005856 | 3,797E-20 | "stearoyl-CoA 9-desaturase" |
| A591_A1381 | *bauA* | -1,051421 | 0,0012576 | "beta-alanine--pyruvate transaminase" |
| A591_A1386 |  | -1,015628 | 0,0015367 | "N-acetylglucosaminylphosphatidylinositol |
| A591_A1387 | *prmA* | -1,116579 | 0,0005139 | "ribosomal protein L11 methyltransferase-like |
| A591_A1388 | *pgaC* | -1,021558 | 0,0011728 | "glycosyltransferase, group 2 family protein" |
| A591_A1394 | *phaC* | 1,783419 | 0,0019288 | "phaC, poly(R)-hydroxyalkanoic acid synthase, class III, |
| A591_A1405 |  | -1,338182 | 0,009195 | "spore coat protein, U domain family" |
| A591_A1548 |  | -1,325008 | 0,0042735 | "putative N-acetyltransferase YedL" |
| A591_A1591 | *fadA* | 1,3786701 | 0,0279507 | "acetyl-CoA C-acyltransferase" |
| A591_A1592 | *kar* | 1,175377 | 0,0248111 | "KR domain protein" |
| A591_A1865 | efflux pump membrane protein | 1,4853965 | 0,0011728 | "efflux pump membrane protein" |
| A591_A2023 |  | -1,069798 | 0,0026296 | "putative N-acetyltransferase YedL" |
| A591_A2027 |  | -1,056854 | 0,0338672 | "putative N-acetyltransferase YedL" |
| A591_A2028 |  | -1,256203 | 0,0078055 | "putative N-acetyltransferase YedL" |
| A591_A2106 |  | -1,091126 | 0,0138812 | "putative N-acetyltransferase YedL" |
| A591_A2115 | *aidA* | 1,2368667 | 0,0032805 | "alpha/beta hydrolase family protein" |
| A591_A2396 | *ywrO* | -1,821392 | 0,0236697 | General stress protein |
| A591_A2397 |  | -1,28627 | 0,0271193 | "putative N-acetyltransferase YedL" |
| A591_A2399 | *katE* | -1,216769 | 7,741E-05 | Catalase KatE-intracellular protease |
| A591_A2403 |  | -1,031448 | 0,0495405 | "putative N-acetyltransferase YedL" |
| A591_A2405 |  | -1,075849 | 0,0112989 | "putative N-acetyltransferase YedL" |
| A591_A2664 | HMA domain | -1,128848 | 0,0024583 | "heavy metal-associated domain protein" |
| A591_A3101 |  | -1,065843 | 0,0240395 | "putative N-acetyltransferase YedL" |
| A591_A3412 | *fadD* | 1,0946636 | 0,0024583 | "AMP-binding enzyme" |
| A591_A3754 | *scd* | 1,5858542 | 9,884E-19 | "stearoyl-CoA 9-desaturase" |
| A591_A3755 |  | 2,2203347 | 2,199E-22 | "oxidoreductase NAD-binding domain protein" |

**Table S2: Transcriptional analysis comparison between *A. baumannii* AB5075 (LB broth) vs *A. baumannii* *Δhns* and AB5075 (LB broth) vs *A. baumannii* AB5075 (LB broth supplemented with HSA)**

| **11 common genes in "LB WT- LB Δnhs" and "LB WT - HSA-WT":** | | | | | | | | |
| --- | --- | --- | --- | --- | --- | --- | --- | --- |
|  |  | function |  | LB WT- LB Δnh | | LB WT - HSA-WT | |  |
| A591_A0345 | ABUW_0374 | QS | *acdA* | 1,7022 | 8,56E-05 | 1,1285 | 4,18E-02 | "acyl-CoA dehydrogenase N-terminal domain protein" |
| A591_A1394 | ABUW_1476 | metabolism | *phaC* | 1,5965 | 1,50E-03 | 1,7834 | 1,93E-03 | "phaC, poly(R)-hydroxyalkanoic acid synthase, class III, |
| A591_A1405 | ABUW_1487 | biofilm | *csuAB* | -5,8326 | 4,23E-54 | -1,3382 | 9,19E-03 | "spore coat protein, U domain family" |
| A591_A1591 | ABUW_1674 | beta oxidation | *fadA* | 1,9695 | 7,68E-05 | 1,3787 | 2,80E-02 | "acetyl-CoA C-acyltransferase" |
| A591_A1592 | ABUW_1675 | QS | *kar* | 1,7278 | 3,07E-05 | 1,1754 | 2,48E-02 | "KR domain protein" |
| A591_A2115 | ABUW_2151 | QQ | *aidA* | 1,2708 | 4,56E-04 | 1,2369 | 3,28E-03 | "alpha/beta hydrolase family protein" |
| A591_A2396 | ABUW_2433 | general stress | *ywrO* | -1,7630 | 1,00E-02 | -1,8214 | 2,37E-02 | General stress protein |
| A591_A2397 | ABUW_2434 |  |  | -2,0152 | 8,83E-06 | -1,2863 | 2,71E-02 | "putative N-acetyltransferase YedL" |
| A591_A2399 | ABUW_2436 | oxidative stress | *katE1* | -1,1213 | 4,97E-05 | -1,2168 | 7,74E-05 | Catalase KatE-intracellular protease |
| A591_A2403 | ABUW_2440 |  |  | -1,4327 | 4,94E-04 | -1,0314 | 4,95E-02 | "putative N-acetyltransferase YedL" |
| A591_A3412 | ABUW_3479 | QS | *fadD* | 1,5231 | 3,45E-07 | 1,0947 | 2,46E-03 | "AMP-binding enzyme" |
